# Speckle Strain Analysis of Left Ventricular Dysfunction in Paediatric Patients with Bicuspid Aortic Valve—A Pilot Study

**DOI:** 10.3390/children11121514

**Published:** 2024-12-13

**Authors:** Amalia Făgărășan, Simina-Elena Ghiragosian-Rusu, Claudiu Ghiragosian, Liliana Gozar, Carmen Suteu, Daniela Toma, Flavia Cristina Al-Akel, Manuela Cucerea

**Affiliations:** 1 Department of Pediatrics III, Faculty of Medicine, George Emil Palade Univ Med Pharm Sci&Technol. of Târgu Mureș, 540142 Târgu Mureș, Romania; amalia.fagarasan@umfst.ro (A.F.); liliana.gozar@umfst.ro (L.G.); carmen.suteu@umfst.ro (C.S.); daniela.toma@umfst.ro (D.T.); 2 Department of Pediatric Cardiology, Emergency Institute for Cardiovascular Diseases and Transplantation of Târgu Mureș, Gheorghe Marinescu Street No. 50, 540136 Târgu Mureș, Romania; cristina.al-akel@umfst.ro; 3 Department of Surgery IV, George Emil Palade Univ Med Pharm Sci&Technol. of Târgu Mureș, 540142 Târgu Mureș, Romania; claudiu.ghiragosian@umfst.ro; 4 Pathophysiology Department, Faculty of Medicine in English, George Emil Palade Univ Med Pharm Sci&Technol. of Târgu Mureș, 540142 Târgu Mureș, Romania; 5 Department of Neonatology, George Emil Palade Univ Med Pharm Sci&Technol. of Târgu Mureș, 540142 Târgu Mureș, Romania; manuela.cucerea@umfst.ro

**Keywords:** bicuspid aortic valve, speckle tracking, left ventricle function, coarctation of the aorta, aortic stenosis, aortic regurgitation, aortopathy

## Abstract

Background/Objectives: Bicuspid aortic valve (BAV) is a prevalent congenital heart defect that continues to present a significant challenge in the management of paediatric patients. The assessment of left ventricle systolic function is typically conducted through the measurement of the left ventricular ejection fraction. Currently, left ventricle global longitudinal strain (LV GLS) is regarded as a more sensitive indicator, enabling the quantitative assessment of global and segmental ventricular function through the determination of myocardial deformation. Methods: A prospective study was conducted between 10 January 2023 and 10 January 2024 in a tertiary paediatric cardiology referral centre. The study enrolled children aged 6 to 17 years with BAV who were undergoing periodic evaluation, as well as a control group. The primary objective was to analyse the systolic function (global and segmental LV) using the classical method (LV EF) and speckle tracking echocardiography (STE). Results: The study group comprised 73 patients with a mean age of 13 years and was predominantly male. The control group comprised 55 patients. The phenotype IB with aortic regurgitation (AR) was the most prevalent. The results of the STE evaluation in the control group demonstrated mean GLS values between −22.1% and −22.8%. A comparison of the BAV group and the control group revealed a significant difference in GLS for the apical four-chamber view (*p* = 0.022). Conclusions: Although the analysis of global LV function demonstrated normal values of EF in patients with BAV, the strain analysis revealed significantly reduced strain in the inferior segment and in the apical four-chamber view, as well as in the anterior segment. Further investigation is required to determine whether reduced LV GLS in paediatric patients with BAV will ultimately result in the development of clinical heart failure. Additionally, it is necessary to ascertain whether this can identify patients with subclinical heart failure and whether early detection can result in a reduction in morbidity.

## 1. Introduction

The bicuspid aortic valve (BAV) represents the most prevalent congenital cardiac anomaly, and it remains challenging to treat in childhood. It can be either undetected or associated with a range of other conditions, including aortic stenosis (AS), aortic regurgitation (AR), coarctation of the aorta (CoA), aortic dilatation, or other congenital heart defects. The progression of these pathologies is variable, and the results published in the literature are still incomplete [1]. BAV may remain asymptomatic, except in the presence of ductal obstructive lesions (severe AS associated with BAV, CoAo, etc.). It is a common occurrence for patients with BAV to experience a deterioration in the functionality of their aortic valve, along with the development of significant aortopathies. In over 50% of cases where this condition is present, surgical intervention is required within 25 years of diagnosis [2,3]. In contrast to the tricuspid aortic valve, the BAV has been observed to produce an abnormal, turbulent flow pattern accompanied by elevated tissue stresses. This phenomenon is most pronounced in the markedly enlarged cusps and at the raphe [4,5].

In the context of AR, this phenomenon results in left ventricular (LV) volume overload as a consequence of the regurgitant flow from the aorta into the LV. This is caused by incomplete aortic valve closure during diastole. AR results in progressive myocardial injury through a number of complex mechanisms, including volume overload of the LV, increased afterload, and myocardial ischaemia. In the event that the compensatory mechanisms for these myocardial injury factors prove inadequate, cardiac failure will ensue, with an unfavourable prognosis. It is therefore recommended that surgical intervention be considered when significant LV enlargement and/or systolic dysfunction are observed in severe AR, in order to circumvent the advent of symptomatic cardiac failure [6].

It is established that a hyperdynamic state occurs in patients with BAV and AS. This must be considered when making a clinical decision regarding the optimal timing of treatment. It is therefore important to analyse the contractile function of the LV. It seems that the longitudinal subendocardial fibres are the first to be impacted by exposure to pressure overload. Consequently, the earliest changes in these fibres are recorded long before alterations in the shortening or ejection fraction become apparent [7].

The LV myocardium is characterised by a complex architecture, comprising circumferential fibres in the mid-wall layer and longitudinal fibres in the endocardial and epicardial layers. In addition, this process leads to the development of irregular and complex contraction patterns, which result in the establishment of long-lasting alterations in myofiber orientation. These changes are observed from right-handed helices in the subendocardium to left-handed helices in the subepicardium. Deformation of the LV is observed to occur in the form of radial thickening, as well as longitudinal and circumferential shortening. Left ventricular ejection fraction (LVEF) represents the standard method for assessing left ventricular systolic function in both adults and children. At present, LV global longitudinal strain (GLS) is regarded as a more sensitive indicator. LV GLS enables a quantitative assessment of global and segmental ventricular function by determining/measuring myocardial deformation, independently of ventricular geometry and angle. It is possible for GLS to be reduced prior to a decline in LV EF being observed. In order to utilise GLS for the assessment of LV function in paediatrics, it is essential to be aware of the typical range of normal values [8,9].

It is also essential to consider the impact of “physiological variation”, which encompasses patient demographic data (age, gender, and clinical factors, such as chest wall conformation, heart rate, blood pressure, and body surface area weight), equipment and technique variables (software, frame rate, and heart rate), and other factors [8,9,10].

With regard to the evaluation of myocardial dysfunction in children, a number of meta-analyses have been conducted with the objective of establishing a threshold. One such meta-analysis is that conducted by Philip T. Levy and colleagues on the normality of LV deformation in paediatric patients. The objective of the authors was to define a range of normal measures of LV deformation. To this end, they collated data from all the studies that reported values for cohorts of paediatric patients deemed to be in a normal state or under control. Accordingly, the mean GLS LV was set at −20.2% (95% CI, −19.5% to −20.8%) in healthy children. In a separate meta-analysis, Jashari et al. determined that the mean normal GLS values ranged from −12.9 to −26.5 (mean, −20.5; 95% CI, −20.0 to −21.0) [11,12].

The findings of multiple studies conducted on adult patients with BAV and associated AS and/or AR, with or without ascending aorta dilatation, respectively, have demonstrated that GLS is an independent predictor of long-term adverse outcomes in patients presenting with minimal symptoms and a preserved EF [13,14].

Although this assessment method is becoming increasingly important for evaluating cardiac dysfunction in children, there is currently a paucity of data on the efficacy of these novel parameters, necessitating further investigation.

## 2. Materials and Methods

A prospective observational analytic study was conducted between 10 January 2023 and 10 January 2024 at a tertiary paediatric cardiology referral centre. The study enrolled children aged 6 to 17 years old with previously known BAV who were undergoing periodic evaluation. Furthermore, a control group of healthy children, matched for anthropometric data, was included in the study.

The exclusion criteria included age below five years, genetic syndromes, metabolic disorders, oncological diagnoses, hepatic diseases, and chronic illnesses of the respiratory or renal systems.

### 2.1. Cardiac Ultrasound Evaluation

All the echocardiograms were conducted using a Philips-EPIQ CVx 3D nSIGHT Plus ultrasound device (Philips, Andover, MA, USA). The acquired images were stored in a digital imaging and communications in medicine (DICOM) format and subsequently analysed offline. The aortic valve phenotype was determined by analysing parasternal short-axis sections. The valve phenotype was classified according to the number of raphes and the anatomical configuration of the cusps. The most recent data on BAV were used to inform the selection of the standard terminology, which was then employed in the analysis [15]. The parasternal long-axis view was employed to calculate Z-scores, perpendicular to the long axis of the aorta, utilising the inner edge technique (aortic annulus, aortic root, sinotubular junction (STJ), and ascending aorta (1 cm distal to the STJ)) [15,16]. In each case, the value of the diameter was obtained by calculating the mean of the diameters measured in three consecutive beats. Furthermore, a Z-score was calculated by correlating body surface area with the aortic annulus, aortic root, STJ, and ascending aorta using the Cantinotti formula [16,17]. The diagnosis of AS was based on the measurement of the peak and mean pressure gradients (calculated using the simplified Bernoulli equation) using continuous wave Doppler ultrasound. The classification of AR was as follows: absent/none, moderate, and severe.

The suprasternal view was employed to observe the aortic arch, its branches, and the descending aorta, with a view to determining whether CoAo was present or absent.

**Figure 1:**
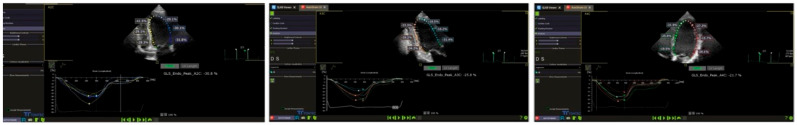
Representative example of left ventricular global longitudinal strain from the study sample.

LV systolic function was evaluated in accordance with the current guidelines for systolic function analysis. Ejection fraction (EF) was calculated using the Teicholz formula in the absence of an asynchrony sign and the modified Simpson method (2D apical four-chamber view). The 2D speckle tracking method was subsequently analysed offline using the left ventricular autostrain functions of the Philips QLAB 15 software (Figure 1). A two-dimensional evaluation, which is typical for the apical 4-, 2-, and 2-chamber view (2D speckle tracking echocardiography—STE), within a frame rate of over 70 Hz, was employed to analyse GLS and left ventricular segmental strain. This was conducted as the interventricular septum and walls were divided into three segments (inferior ventricular—IV basal, IV medial, IV apical; left ventricular—LV basal, LV medial, LV apical). Subsequently, the aforementioned parameters were subjected to a comparative analysis between the two study groups [16].

### 2.2. Statistical Analysis

The statistical analysis was conducted using the R statistical computing platform version 4.4.2 [18], and the graphical representations were generated with the matplotlib Python version 4.4.2 library for data visualisation. The categorical data were presented as a number and percentage, while continuous parametric data were expressed as the mean ± standard deviation, and non-parametric continuous data as the median (interquartile range). The assumption of normality was evaluated through the implementation of the Kolmogorov–Smirnov test. Comparisons of central tendency were conducted using *t*-tests for parametric data and Mann–Whitney tests for non-parametric data. In instances where multiple groups were involved, a one-way ANOVA was employed for parametric data, with post hoc Tukey tests subsequently conducted. For non-parametric data, a Kruskal–Wallis test was utilised, with Dunn’s test employed for post hoc analysis. Frequency comparisons were performed using variations of the chi-square test. A significance level of α = 0.05 was considered for all analyses.

### 2.3. Ethics

The research was conducted in accordance with the principles set forth in the Declaration of Helsinki. Prior to inclusion in the study, the research team obtained a signed informed consent form from at least one of the legal tutors of each child. The research protocol was approved by the Ethics Committee of the Târgu Mureș Emergency Institute for Cardiovascular Diseases and Transplantation (approval no. 8902/20 December 2022) and by that of the George Emil Palade University of Medicine, Pharmacy, Science, and Technology of Târgu Mureș (approv. no. 2034/26 January 2023).

### 2.4. Objectives

The primary objective of this study was to analyse the systolic function (global and segmental LV) by the classical method (LV EF) and by speckle tracking in order to quantify the degree of LV dysfunction in the BAV and control groups. The objective was to analyse the capacity of the aforementioned factors to discriminate between the groups and their relationship with the degree of valvular regurgitation, AS, aortopathy and CoA. The objective of this review was to enhance comprehension of the contributions of global longitudinal strain (GLS) to the evaluation and management of patients with BAV in clinical practice.

### 2.5. Definitions

FE—this was calculated using the Teicholz formula (in the absence of an asynchrony sign, short axis) and the modified Simpson method (2D aA4C—apical four-chamber view).

LV systolic LV dysfunction, based on 2D speckle tracking echocardiography, was defined as an LVGLS value of less than −20.2%, which is consistent with the 2016 meta-analysis by Levy and colleagues [16].

The aortic bicuspid phenotypes were classified as follows: fused BAV (IA-right–left cusp fusion, IB-right non-cusp fusion, IC-left non-cusp fusion, and indeterminate phenotypes); 0-sinus BAV (laterolateral—0 LL and anteroposterior—0 AP phenotypes); and II-partial-fusion BAV and/or mild BAV forms (small raphe, single phenotype) [19].

AS was classified in accordance with the recommendations set forth in the guideline, as follows: mild (mean gradient < 20 mm Hg and peak velocity < 3 m/s), moderate (mean gradient between 20 and 40 mm Hg), and severe (mean gradient > 40 mm Hg and peak valve velocity > 4 m/s) [19,20].

The classification of AR was based on the ratio of the jet width to the annular diameter, which was assessed in the parasternal long-axis view. Additionally, the presence of descending AR was determined through colour Doppler imaging. Furthermore, left ventricular dimensions were evaluated and categorised as mild-normal, moderate-normal, dilated, or severe-dilated. Finally, the jet deceleration rate was considered, with values of CW (PHT pressure half-time, ms) and categorised as slow (less than 500), moderate (between 500 and 200), or severe (less than 200) [19,21].

Aortopathy is defined, by convention, as the presence of an aortic root and/or ascending aorta dilatation with a Z-score greater than two standard deviations (SD) [22].

Pro-BNP is considered normal at a cutoff of less than 178 ng/L for children aged between 1 and 19 years [23].

## 3. Results

The descriptive data and a comparison of the baseline characteristics are presented in Table 1.

**Figure 2:**
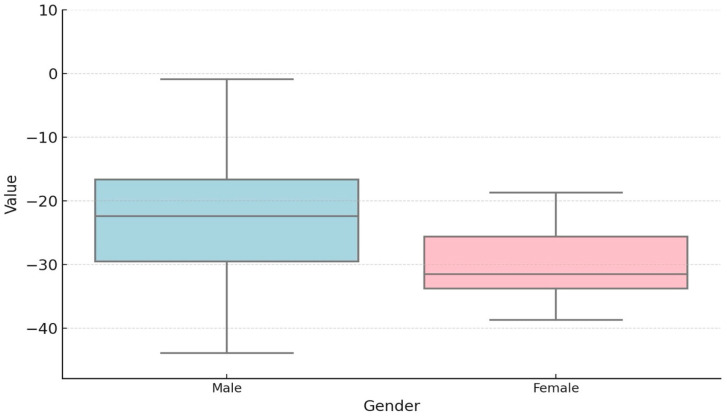
Gender distribution of BAV patients (BAV group—75 patients, male—55 patients, 75.34%, female—18 patients, 24.65%).

The study group comprised 73 patients with a mean age of 13 years and was predominantly male (Figure 2).

No significant differences were identified between the BAV and control groups with regard to age, weight, body surface area, or cardiac blood pressure. The results of the serum biomarker analysis for cardiac dysfunction (pro-BNP) demonstrated no changes indicative of dysfunction (the mean values were 13.80 ng/L, compared to the reference cutoff of 178 ng/L).

**Figure 3:**
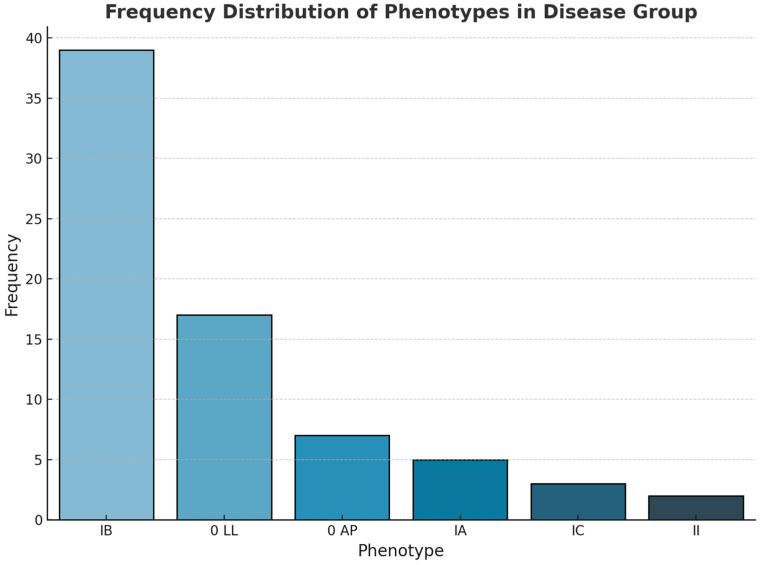
Frequency distribution of phenotypes in BAV group (BAV—bicuspid aortic valve).

With regard to the analysis of the BAV phenotype, the results indicate that 53.42% of the patients exhibited type IB, followed by phenotype O, IA, IC, or II in 32.87%, 6.8%, 4.1, and 2.7% of cases, respectively (see Figure 3).

An additional element of analysis was the classification of the valvulopathy (insufficiency or regurgitation) in the BAV group. As illustrated in Table 2, the findings indicate that 68.49% of the BAV cases exhibited mild AR.

With regard to the presence of aortic dilatation (aortopathy), the results of our study indicate that the study group (BAV group) exhibited significantly higher absolute values for aortic root and ascending aorta size than the control group (see Table 3).

The primary objective of the study was to quantify the degree of global and/or segmental VS dysfunction. The results demonstrated that the global contractile function, as quantified by EF and fractional shortening (FS), was comparable to that of the control group, with a mean value of 67%. This indicates that no global contractile dysfunction was identified (see Table 1).

The results of the STE evaluation in the control group indicate mean GLS values ranging from −22.1% to −22.8% for the global evaluation and from −18.6% to −29.15% for the segmental evaluation (Table 4).

A comparison of the BAV group and the control group revealed a significant difference in GLS for the A4C (*p* = 0.022). Moreover, the regional longitudinal strain was found to be significantly lower in the BAV group in the inferior segment (*p* = 0.04, −17.13%) and apical anteromedial (*p* = 0.03, −16.03%) from the A4C view, as well as in the anterior segment (*p* = 0.02, −22.73%). The results are presented in Table 5.

The potential influence of gender on the degree of segmental dysfunction in the study group was also investigated, with no statistically significant differences identified (Table 6).

The degree of segmental dysfunction by STE was analysed between the control group and the BAV group, which was divided into the following subgroups. Statistically significant results were observed in the comparisons between BAV and AR, BAV and AS, BAV and CoAo, and BAV and aortopathy (see Table 7).

To gain further insights from the data obtained, a post hoc analysis was conducted between the GLS A4C values of the lower SIV and anteromedial SIV segments, respectively, and the GLS A2C of the anteromedial SIV segment. This analysis was conducted between the control and BAV groups, which were divided according to their associated pathologies. The results of this analysis are presented in Table 8.

A comparison of GLS values by bicuspid phenotype was conducted in the BAV group; however, no statistically significant results were yielded. In contrast, the results of the segmental evaluation yielded statistically significant outcomes (Table 9).

A post hoc analysis of the dataset revealed the presence of statistically significant data between the mean values observed in the inferolateral segment of the IVS in the A3C, OLL, and IA phenotypes with *p*-values of 0.02, IA and IB with *p*-values of 0.05, and IA and IC with *p*-values of 0.05 (Table 10).

With regard to the anteromedial segment of the IVS from the A3C incidence, no statistically significant data were obtained.

## 4. Discussion

Apart from the fact that an increased prevalence of this valvular pathology has been observed within the wider population, BAV is a significant cause of paediatric morbidity as it often coexists with a variety of valvular and aortopathy complexes. These may include AS/AR, aortic dilatation, or a combination of these conditions. They often manifest at different stages of childhood, and their association with infective endocarditis is relatively infrequent [19,24,25,26,27]. As has been demonstrated in the majority of studies conducted in both paediatric and adult pathology, BAV is the most prevalent in males. This finding is supported by the results of our study. The mean age of the subjects in the study was 13 years, with no significant difference between the two groups. In patients with BAV, both leaflets experience a considerable degree of stress overload, particularly at the site of fusion, which is a factor in the accelerated degeneration of the valve [26].

The morphology of the valve may serve as an indicator of potential issues related to stenosis, insufficiency, or both. In a comprehensive multicentre retrospective study (MIBAVA Consortium) encompassing over 2000 children with BAV (mean age 10.2 years), the most prevalent morphology was identified as IA fusion (65.7%), followed by IB fusion (32.9%) [26]. The IB phenotype was identified as the most prevalent, occurring in both patients with AS/AR and those with CoA. Some studies have demonstrated that paediatric patients with left heart obstructive lesions are more frequently observed to present a right-to-left (R-L) cusp fusion [19,26]. Additionally, the MIBAVA Consortium study demonstrated that R-L fusion was linked to the occurrence of aortic coarctation, whereas R-N fusion was associated with the onset of valve dysfunction, including stenosis and/or regurgitation. In the present study, the most frequently observed valve dysfunction was mild AR. The morphology of BAV is an important factor that could directly influence the dilatation of the aorta. It has been demonstrated in previous studies that the IB phenotype is most often associated with dilatation of the ascending aorta and is usually associated with aortic insufficiency. This was also demonstrated in the present study, in which 30 patients had aortopathy; of these, 14 had the IB phenotype. The literature reports indicate that the IB phenotype is associated with a poorer prognosis in terms of the progression of valvular lesions (As/AR), which is a crucial consideration in the follow-up of paediatric patients with BAV [28]. The Z-scores of children with BAV appear to be higher at the site of the annulus, sinus of Valsalva, sinotubular junction, and ascending aorta [29]. The present study revealed statistically significant differences between the absolute size and Z-scores of the aortic annulus, sinus of Valsalva, sinotubular junction, and ascending aorta. Proximal ascending aortic dilatation is a common finding in paediatric and adolescent patients with BAV, occurring in approximately 50% of cases. It is hypothesised that haemodynamic and genetic factors may explain the link with aortic disease. The MIBAVA Consortium study demonstrated a correlation between R-N fusion and an enlarged aorta, indicating a potential genetic influence. In other instances, haemodynamic factors appear to be implicated, with AR linked to wider sinotubular junction diameters [26,30]. Aortic dilatation appears to progress at a relatively slow rate during childhood. In a recent series [31], a low rate of progression was substantiated during the infantile and adolescent phases, which are distinguished by pronounced somatic growth. The mean increase in the aortic root and ascending aorta was 1.00 mm per year. The prevalence and advancement of aortic dilatation are less frequent in BAV associated with aortic coarctation compared to isolated BAV. Furthermore, the presence of AR has been associated with larger proximal ascending aortic diameters [16].

Regarding BAV associated with valvular lesions, studies have shown that valvular dysfunctions—such as severe or moderate stenosis and severe or moderate regurgitation—occurring in childhood are significant predictors of aortopathy and its progression. Furthermore, patients with combined mild stenosis and regurgitation have been found to have a ninefold increased risk of developing significant aortic dilatation in early adulthood. This indicates that haemodynamic factors exert a significant influence [32]. Given the distinctive aortic morphology, the use of specific aortic reference nomograms for children and adolescents with BAV may prove beneficial in monitoring this progression [26,27].

In relation to the primary objective of the study, speckle analysis of the extent of segmental dysfunction serves as a valuable tool for the early recognition of subclinical dysfunction in the LV, extending beyond the limitations associated with reference values based on age group, gender, and the experience of the investigator. The data from the literature highlight the significance of this parameter in a range of congenital and acquired heart diseases in children [33,34]. The analysis of healthy children revealed that the GLS values of the left ventricle ranged from −19.95% to −24.00% (mean: −22.10%); these values were comparable to those obtained in the meta-analysis conducted by Levy et al. and Jashari et al. The mean values of left ventricular deformation, as determined by segmental speckle evaluation, were as follows: basal (−18.85, −21.50 and −27.60), mid-basal (−22.92, −25.77 and −23.8), and apical (−24.72, −24.7 and −22.62). The mean GLS of the A4C was −20.49% for the BAV group, with a mean EF of 67%.

The assessment of segmental VS deformity revealed statistically notable discrepancies between the two groups at the segmental level, specifically in the basal and medial anterolateral segments in A4C and the anteromedial segment in A2C. In an analysis of aortic strain in adults with BAV, Carlos et al. [35] demonstrated that LV GLS was reduced, indicating subclinical impaired LV contraction. This outcome is in accordance with the results of previous studies that have demonstrated alterations in LV mechanics in BAV patients, with a reduction in longitudinal, circumferential, and radial strains, even in the presence of mild valvular disease or in the absence of AS, AR, or aortopathy [35]. Impaired GLS is more common in cases of valvular dysfunction and is linked to the risk of aortic valve replacement, even in BAV patients [26,36]. The results of our study indicate that individuals with AS exhibit reduced GLS values. Previous research has demonstrated that AS is associated with diminished values of the inferoseptal or anteroseptal wall of the LV base and the midportion of the LV. These findings are consistent with those of the present study. The observed reductions in GLS are attributed to ischemia and lesions, as evidenced by 2D strain analysis [36]. Myocardial fibrosis is an early phenomenon in the natural history of AS, with the potential to impair both the systolic and diastolic function of the heart. It provides a substrate that is conducive to the emergence of ventricular arrhythmias and is involved in the development of heart failure and sudden cardiac death. These findings suggests that the present echocardiographic evaluation of LV function based on the LVEF quantification alone is insufficient. It is thus necessary to identify new markers for the detection of subtle myocardial impairment; this is required in order to enhance risk stratification and outcome prediction in patients with AS [37]. In adults, altered GLS has been observed in asymptomatic patients presenting with severe AS and preserved EF; these patients are at an elevated risk of developing symptoms and require surgical intervention [26,38]. There is mounting evidence to suggest that GLS has a prognostic role in asymptomatic patients with AS. The American Society of Echocardiography has acknowledged the additional value of LV GLS over traditional LVEF measurements and has recommended its clinical use in patients [38,39].

It is becoming increasingly recognised as a means of guiding the management of valvular heart disease. In AR, the longitudinal orientation of myocardial fibres in the subendocardial layer results in decreased longitudinal contraction, which is an early indicator of LV dysfunction [40]. In patients with asymptomatic moderate AR and preserved ejection fraction, reduced global longitudinal strain is associated with an increased risk of mortality in those who did not undergo aortic surgery.

It is noteworthy that the cohort of patients with coarctation and BAV exhibited lower GLS values. Of the patients with CoAo, nine had undergone surgical treatment, while two were not yet indicated for surgical or interventional treatment. In a prospective study of adults with CoA who had undergone surgical treatment, Myrthe E. Menting and colleagues observed that LV GLS values were lower compared with the control group (−17.1% ± 2.3% vs. −20.2% ± 1.6%, *p* < 0.001). The results of that study describe a 70% BAV level in patients with CoA. For this category of patients, the literature is limited, and further studies are needed [41].

Further investigation is required to determine whether reduced LV GLS in the paediatric population with BAV ultimately results in clinical heart failure and whether it serves as a marker for identifying patients with subclinical heart failure. Additionally, the potential of early detection to reduce morbidity warrants further study. Therefore, GLS may assist in risk stratification, enabling the identification of the optimal timing for treatment (surgical or interventional) [26].

## 5. Limitations

It is important to note that our study is subject to several limitations. Firstly, it was conducted at a single centre with a relatively small sample size. The study cohort comprised children with an average age of 13 years. However, the period of the newborn and infants was not analysed. The investigation did not extend to the measurement of circumferential and radial strain of the LV for comparison. A further limitation of the study was the lack of evaluation of intra-observer reproducibility.

The study is further limited by the lack of standardisation, the use of Z-scores to analyse aortopathy, which may result in changes in the reference values, and the limitations of 2D imaging in terms of image quality, artefacts, image dropout, and frame rate.

Another limitation was the presence of patients with CoA, but it should be noted that in this group, nine patients had had an operation and did not have a significant residual gradient, and the two patients who had not had an operation did not have an indication for surgical/interventional treatment at the time of evaluation. Therefore, in these areas, further long-term, multicentre, multi-arm studies with larger groups are required. The present study was merely observational and thus requires comparison with a meta-analysis to provide a comprehensive understanding of the subject matter.

## 6. Conclusions

Despite the normal values of EF and FS observed in the global LV function analysis of patients with BAV, the strain analysis revealed significantly reduced values in the inferior segment and in the apical four-chamber view, as well as in the anterior segment.

The most prevalent phenotype was IB, which was observed in conjunction with mild AR.

Further investigation is required to determine whether reduced LV GLS in the paediatric population with BAV ultimately results in clinical heart failure and whether it serves as a marker for identifying patients with subclinical heart failure.

## Figures and Tables

**Table 1:** Baseline characteristics of the study sample (BSA—body surface area, BMI—body mass index, BP—blood pressure, HR—heart rate, EF—ejection fraction, SF—shorten fraction).

Parameter	Control Group (n = 54)	BAV Group (n = 73)	*p*-Value
Age (years)	13.00 (10.00–15.00)	13.00 (9.50–16.00)	0.928
Weight (kg)	45.50 (36.50–57.75)	53.00 (32.50–66.00)	0.285
BSA (m^2^)	1.41 ± 0.31	1.49 ± 0.41	0.204
Vitamin D (ng/mL)	26.40 (19.64–35.90)	30.16 (20.99–54.92)	0.29
pro-BNP (ng/L)	10.90 (10.00–20.25)	13.80 (10.00–21.60)	0.292
BMI (kg/m^2^)	18.98 ± 3.53	19.78 ± 4.41	0.274
Systolic BP (mmHg)	110.00 (98.00–120.75)	111.00 (104.00–121.00)	0.4
Diastolic BP (mmHg)	66.50 (60.00–72.00)	65.00 (60.00–70.00)	0.65
HR (b/min)	83.57 ± 13.51	77.23 ± 14.39	0.017
EF (mean, SD)	67.29 (7.917)	66.64 (7.708)	0.3936
SF (mean, SD)	38.18 (7.52)	36.63 (6.799)	0.1859

**Table 2:** Frequency distribution of associated pathologies in BAV group (CoAo—coarctation of the aorta, BAV—bicuspid aortic valve, AS—aortic stenosis, AR—aortic regurgitation).

Pathology	BAV Group (n = 73)
Unoperated CoAo, n (%)	2 (2.74)
Operated CoAo, n (%)	9 (12.33)
Mild AS, n (%)	22 (30.14)
Moderated AS, n (%)	8 (10.96)
Severe AS, n (%)	8 (10.96)
Mild AR, n (%)	50 (68.49)
Moderated AR, n (%)	11 (15.07)
Severe AR, n (%)	2 (2.74)
Aortopathy, n (%)	30 (41.10)

**Table 3:** Comparison between absolute size and Z-score of aortic annulus, sinus of Valsalva, sinotubular junction, and ascending aorta of BAV group and control group (BAV—bicuspid aortic valve).

Parameters	BAV Group (n = 73)	Group Control (n = 55)	*p* Value
Aortic valve annulus (mm, mean, SD)	22.22 (4.35)	23.45 (27.83)	0.0010 **
Aortic valve annulus Z-score (mean, SD)	1.67 (1.47)	3.13 (19.30)	<0.0001 **
Sinus of Valsalva (mm, mean, SD)	25.56 (5.62)	26.83 (31.36)	0.0023 **
Sinus of Valsalva Z-score (mean, SD)	0.521 (1.729)	−0.648 (1.207)	0.0001 *
Sinotubular junction (mm, mean, SD)	23.41 (6.714)	20.08 (6.007)	0.0065 **
Sinotubular junction Z-score (mean, SD)	1.735 (1.531)	0.605 (0.994)	<0.0001 **
Ascending aorta (mm, mean, SD)	30.95 (26.41)	18.50 (7.927)	<0.0001 **
Ascending aorta Z-score (mean, SD)	1.881 (1.992)	−0.437 (1.097)	<0.0001 **

* Student *t* test; ** Mann–Whitney U test.

**Table 4:** Results of global and segmental longitudinal strain in control group (abbreviations: BIS—basal inferoseptal, MIS—mid-inferoseptal, AIS—apical inferoseptal, AAL—apical anterolateral, MAL—mid-anterolateral, BAL—basal anterolateral, BI—basal inferior, MI—mid-inferior, AI—apical inferior, AA—apical anterior, MA—mid-anterior, BA—basal anterior, BIL—basal inferolateral, MIL—mid-inferolateral, AIL—apical inferolateral, AAS—apical anteroseptal, MAS—mid-anteroseptal, BAS—basal anteroseptal).

Parameters	Control Group (n = 55)
GLS A4C (mean, SD)	−21.25 (7.17)
BIS (mean, SD)	−19.46 (6.82)
MIS (mean, SD)	−22.92 (5.04)
AIS (mean, SD)	−24.72 (7.20)
AAL (mean, SD)	−21.53 (7.23)
MAL (mean, SD)	−19.48 (6.70)
BAL (mean, SD)	−27.80 (8.49)
GLS A2C (mean, SD)	−21.52 (9.07)
BI (mean, SD)	−22.13 (8.14)
MI (mean, SD)	−25.77 (5.89)
AI (mean, SD)	−24.94 (7.19)
AA (mean, SD)	−18.60 (9.23)
MA (mean, SD)	−21.15 (5.18)
BA (mean, SD)	−29.15 (8.42)
GLS A3C (mean, SD)	−22.73 (4.01)
BIL (mean, SD)	−27.52 (12.44)
MIL (mean, SD)	−22.40 (7.87)
AIL (mean, SD)	−22.86 (10.92)
AAS (mean, SD)	−22.62 (10.41)
MAS (mean, SD)	−21.63 (7.53)
BAS (mean, SD)	−19.79 (10.42)

**Table 5:** GLS correlations between BAV group and control group (abbreviations: BIS—basal inferoseptal, MIS—mid-inferoseptal, AIS—apical inferoseptal, AAL—apical anterolateral, MAL—mid-anterolateral, BAL—basal anterolateral, BI—basal inferior, MI—mid-inferior, AI—apical inferior, AA—apical anterior, MA—mid-anterior, BA—basal anterior, BIL—basal inferolateral, MIL—mid-inferolateral, AIL—apical inferolateral, AAS—apical anteroseptal, MAS—mid-anteroseptal, BAS—basal anteroseptal).

Parameter	BAV Group (n = 73)	Control Group (n = 55)	*p*
GLS A4C (mean, SD)	−20.49 (4.89)	−21.25 (7.17)	0.0229 **
BIS (mean, SD)	−17.13 (8.21)	−19.46 (6.82)	0.0430 **
MIS (mean, SD)	−21.69 (6.52)	−22.92 (5.04)	0.3220 *
AIS (mean, SD)	−25.24 (9.55)	−24.72 (7.20)	0.7700 *
AAL (mean, SD)	−21.49 (9.08)	−21.53 (7.23)	0.9819 *
MAL (mean, SD)	−16.03 (8.45)	−19.48 (6.70)	0.0336 *
BAL (mean, SD)	−25.56 (12.24)	−27.80 (8.49)	0.4213 **
GLS A2C (mean, SD)	−21.65 (4.96)	−21.52 (9.07)	0.2907 **
BI (mean, SD)	−18.79 (8.64)	−22.13 (8.14)	0.0742 **
MI (mean, SD)	−24.92 (7.17)	−25.77 (5.89)	0.5700 *
AI (mean, SD)	−28.03 (8.05)	−24.94 (7.19)	0.0678 *
AA (mean, SD)	−20.23 (8.70)	−18.60 (9.23)	0.4010 *
MA (mean, SD)	−17.60 (9.09)	−21.15 (5.18)	0.0407 **
BA (mean, SD)	−22.73 (12.69)	−29.15 (8.42)	0.0231 **
GLS A3C (mean, SD)	−21.80 (5.24)	−22.73 (4.01)	0.3078 **
BIL (mean, SD)	−26.64 (11.71)	−27.52 (12.44)	0.7342 *
MIL (mean, SD)	−20.46 (9.26)	−22.40 (7.87)	0.3154 *
AIL (mean, SD)	−24.09 (8.68)	−22.86 (10.92)	0.5515 *
AAS (mean, SD)	−24.28 (11.73)	−22.62 (10.41)	0.5041 *
MAS (mean, SD)	−19.79 (7.47)	−21.63 (7.53)	0.3730 **
BAS (mean, SD)	−17.07 (8.97)	−19.79 (10.42)	0.1891 *

* Student *t* test; ** Mann–Whitney U test.

**Table 6:** Correlation of GLS values according to patient gender in the BAV group (abbreviations: BIS—basal inferoseptal, MIS—mid-inferoseptal, AIS—apical inferoseptal, AAL—apical anterolateral, MAL—mid-anterolateral, BAL—basal anterolateral, BI—basal inferior, MI—mid-inferior, AI—apical inferior, AA—apical anterior, MA—mid-anterior, BA—basal anterior, BIL—basal inferolateral, MIL—mid-inferolateral, AIL—apical inferolateral, AAS—apical anteroseptal, MAS—mid-anteroseptal, BAS—basal anteroseptal).

Parameter	Male (n = 55)	Female (n = 18)	*p*-Value
GLS A4C (mean, SD)	−20.10 ± 5.27	−21.60 ± 3.66	0.193
BIS (mean, SD)	−14.65 (−19.73–−11.12)	−18.25 (−21.72–−16.02)	0.016
MIS (mean, SD)	−21.14 ± 6.87	−23.29 ± 5.42	0.185
AIS (mean, SD)	−26.28 ± 10.15	−22.22 ± 7.30	0.075
AAL (mean, SD)	−22.71 ± 9.15	−17.97 ± 8.41	0.052
MAL (mean, SD)	−15.19 ± 8.07	−18.46 ± 9.48	0.202
BAL (mean, SD)	−24.15 ± 11.87	−29.66 ± 13.06	0.126
GLS A2C (mean, SD)	−21.18 ± 4.90	−23.01 ± 5.15	0.201
BI (mean, SD)	−16.55 (−22.25–−12.95)	−20.40 (−26.08–−18.52)	0.061
MI (mean, SD)	−25.03 ± 7.35	−24.62 ± 7.04	0.837
AI (mean, SD)	−28.28 ± 8.34	−27.30 ± 7.60	0.648
AA (mean, SD)	−20.61 ± 9.15	−19.16 ± 7.68	0.516
MA (mean, SD)	−17.90 (−22.07–−13.45)	−18.80 (−24.12–−17.35)	0.183
BA (mean, SD)	−22.40 (−29.52–−16.65)	−31.55 (−33.80–−25.62)	0.019
GLS A3C (mean, SD)	−21.91 ± 5.18	−21.48 ± 5.67	0.78
BIL (mean, SD)	−26.87 ± 12.07	−25.97 ± 11.25	0.777
MIL (mean, SD)	−17.75 (−25.68–−14.47)	−22.25 (−25.03–−17.65)	0.323
AIL (mean, SD)	−24.32 ± 8.61	−23.43 ± 9.35	0.725
AAS (mean, SD)	−24.55 ± 10.86	−23.49 ± 14.54	0.78
MAS (mean, SD)	−19.40 (−24.10–−15.45)	−19.85 (−21.03–−17.48)	0.995
BAS (mean, SD)	−16.66 ± 8.42	−18.27 ± 10.82	0.571

**Table 7:** Comparison of the GLS values between the control group and the BAV group according to the associated pathology (abbreviations: BIS—basal inferoseptal, MIS—mid-inferoseptal, AIS—apical inferoseptal, AAL—apical anterolateral, MAL—mid-anterolateral, BAL—basal anterolateral, BI—basal inferior, MI—mid-inferior, AI—apical inferior, AA—apical anterior, MA—mid-anterior, BA—basal anterior, BIL—basal inferolateral, MIL—mid-inferolateral, AIL—apical inferolateral, AAS—apical anteroseptal, MAS—mid-anteroseptal, BAS—basal anteroseptal).

Parameter	Group 1—Control Group (n = 54)	Group 2—AS (n = 38)	Group 3—CoA (n = 11)	Group 4—Aop (n = 30)	Group 5—AR (n = 61)	*p*-Value
GLS A4C (mean, SD)	**−22.10 (−24.00–−19.95)**	−19.85 (−21.75–−17.43)	−17.90 (−21.40–−16.25)	−20.90 (−22.35–−17.40)	−20.90 (−23.55–−17.85)	0.1
BIS (mean, SD)	−18.85 (−23.58–−15.57)	−15.15 (−19.02–−11.12)	−15.00 (−17.95–−13.40)	−13.70 (−18.25–−9.90)	−16.20 (−20.20–−11.60)	0.022
MIS (mean, SD)	−22.92 ± 5.10	−21.84 ± 6.87	−21.17 ± 7.96	−21.37 ± 7.25	−21.98 ± 6.35	0.874
AIS (mean, SD)	−24.72 ± 7.30	−24.59 ± 8.42	−27.76 ± 10.68	−24.75 ± 10.63	−25.74 ± 10.13	0.854
AAL (mean, SD)	−21.53 ± 7.33	−21.67 ± 9.09	−23.98 ± 9.19	−22.89 ± 9.66	−21.93 ± 9.45	0.917
MAL (mean, SD)	−19.48 ± 6.79	−14.97 ± 8.55	−10.32 ± 3.87	−16.35 ± 9.88	−16.64 ± 8.75	0.018
BAL (mean, SD)	−26.95 (−30.80–−21.73)	−26.15 (−34.08–−18.57)	−21.80 (−31.90–−6.50)	−26.80 (−33.15–−20.75)	−26.60 (−34.45–−18.20)	0.792
GLS A2C (mean, SD)	−22.10 (−25.65–−19.70)	−20.75 (−24.32–−18.75)	−19.70 (−22.10–−18.90)	−21.80 (−25.20–−19.00)	−21.90 (−25.20–−19.00)	0.368
BI (mean, SD)	−21.50 (−25.40–−16.35)	−18.40 (−21.55–−12.78)	−16.00 (−22.00–−11.85)	−18.30 (−22.25–−13.10)	−19.10 (−23.75–−13.60)	0.217
MI (mean, SD)	−25.77 ± 5.99	−24.25 ± 7.16	−22.79 ± 7.27	−25.50 ± 8.20	−25.82 ± 6.92	0.622
AI (mean, SD)	−24.70 (−27.25–−22.15)	−27.05 (−31.10–−21.53)	−27.10 (−33.40–−23.20)	−27.80 (−33.20–−22.70)	−27.80 (−34.05–−23.05)	0.213
AA (mean, SD)	−18.60 ± 9.38	−18.96 ± 7.70	−17.52 ± 4.89	−21.03 ± 7.31	−20.57 ± 8.68	0.559
MA (mean, SD)	−21.20 (−24.30–−16.95)	−17.55 (−20.35–−13.45)	−17.50 (−21.50–−14.85)	−17.50 (−20.90–−12.00)	−18.00 (−23.05–−15.75)	0.039
BA (mean, SD)	−29.15 ± 8.56	−24.34 ± 9.50	−23.53 ± 7.70	−24.71 ± 9.14	−24.61 ± 9.33	0.152
GLS A3C (mean, SD)	−22.80 (−24.35–−20.40)	−20.15 (−23.88–−16.88)	−20.20 (−21.70–−17.05)	−22.00 (−24.75–−18.45)	−21.40 (−26.05–−18.70)	0.286
BIL (mean, SD)	−27.52 ± 12.64	−25.22 ± 11.87	−25.39 ± 11.83	−25.24 ± 12.93	−26.76 ± 12.08	0.921
MIL (mean, SD)	−23.80 (−27.60–−17.90)	−18.15 (−25.00–−13.68)	−19.50 (−27.90–−17.10)	−16.60 (−25.15–−11.60)	−21.70 (−25.75–−15.15)	0.288
AIL (mean, SD)	−22.86 ± 11.10	−23.34 ± 9.21	−17.95 ± 7.74	−24.16 ± 8.63	−24.19 ± 9.00	0.355
AAS (mean, SD)	−22.62 ± 10.58	−23.59 ± 12.27	−24.35 ± 13.61	−28.31 ± 11.29	−24.79 ± 11.77	0.427
MAS (mean, SD)	−21.00 (−24.60–−15.30)	−18.90 (−21.77–−15.50)	−20.50 (−21.30–−12.70)	−19.70 (−25.30–−17.15)	−19.70 (−24.60–−17.10)	0.587
BAS (mean, SD)	−21.10 (−26.65–−13.45)	−15.15 (−19.93–−10.20)	−13.20 (−16.90–−9.65)	−13.70 (−19.15–−9.85)	−16.80 (−21.45–−10.80)	0.198

**Table 8:** Post hoc analysis between control and BAV groups according to the associated pathology (A4C—apical four-chamber view, A2C—apical two-chamber view, BIS—basal inferoseptal, MAL—mid-anterolateral, MA—mid-anterior).

Post Hoc Analysis	
BIS A4C—(Mann–Whitney U)	*p*-value
Group 1—Control group vs. Group 2—AS	0.014
Group 1—Control group vs. Group 3—CoA	0.058
Group 1—Control group vs. Group 4—Aortopathy	0.003
Group 1—Control group vs. Group 5—AR	0.043
Group 2—AS vs. Group 3—CoA	0.95
Group 2—AS vs. Group 4—Aortopathy	0.445
Group 2—AS vs. Group 5—AR	0.427
Group 3—CoAo vs. Group 4—Aortopathy	0.499
Group 3—CoAo vs. Group 5—AR	0.617
Group 4—Aortopathy vs. Group 5—AR	0.117
MAL A2C (Mann–Whitney U)	
Group 1—Control group vs. Group 2—AS	0.007
Group 1—Control group vs. Group 3—CoA	0.067
Group 1—Control group vs. Group 4—Aortopathy	0.008
Group 1—Control group vs. Group 5—AR	0.082
Group 2—AS vs. Group 3—CoA	0.92
Group 2—AS vs. Group 4—Aortopathy	0.77
Group 2—AS vs. Group 5—AR	0.247
Group 3—CoAo vs. Group 4—Aortopathy	0.949
Group 3—CoAo vs. Group 5—AR	0.401
Group 4—Aortopathy vs. Group 5—AR	0.191
MAL A4C (Tukey test HSD)	
Group 1—Control group vs. Group 2—AS	0.137
Group 1—Control group vs. Group 3—CoA	0.013
Group 1—Control group vs. Group 4—Aortopathy	0.563
Group 1—Control group vs. Group 5—AR	0.468
Group 2—AS vs. Group 3—CoA	0.013
Group 2—AS vs. Group 4—Aortopathy	0.563
Group 2—AS vs. Group 5—AR	0.468
Group 3—CoAo vs. Group 4—Aortopathy	0.563
Group 3—CoAo vs. Group 5—AR	0.468
Group 4—Aortopathy vs. Group 5—AR	0.468

**Table 9:** Comparison of the GLS values between the control group and the BAV group according to the phenotype (abbreviations: BIS—basal inferoseptal, MIS—mid-inferoseptal, AIS—apical inferoseptal, AAL—apical anterolateral, MAL—mid-anterolateral, BAL—basal anterolateral, BI—basal inferior, MI—mid-inferior, AI—apical inferior, AA—apical anterior, MA—mid-anterior, BA—basal anterior, BIL—basal inferolateral, MIL—mid-inferolateral, AIL—apical inferolateral, AAS—apical anteroseptal, MAS—mid-anteroseptal, BAS—basal anteroseptal).

Parameter	0 AP	0 LL	IA	IB	IC	*p*-Value
GLS A4C (mean, SD)	−17.51 ± 5.95	−19.46 ± 2.63	−17.70 ± 6.25	−21.48 ± 5.21	−20.87 ± 3.10	0.245
BIS (mean, SD)	−13.70 (−20.45–−10.95)	−15.50 (−18.50–−11.12)	−16.55 (−21.70–−11.88)	−17.40 (−20.25–−11.97)	−18.20 (−24.80–−16.25)	0.43
MIS (mean, SD)	−21.63 ± 8.47	−22.82 ± 6.51	−18.05 ± 7.32	−21.43 ± 6.53	−20.07 ± 2.27	0.483
AIS (mean, SD)	−22.27 ± 12.65	−25.11 ± 5.13	−19.98 ± 13.05	−26.51 ± 10.33	−23.17 ± 6.93	0.982
AAL (mean, SD)	−23.83 ± 10.38	−19.23 ± 6.85	−20.80 ± 7.82	−22.07 ± 10.41	−21.57 ± 5.42	0.869
MAL (mean, SD)	−11.50 (−17.00–−9.25)	−14.30 (−18.88–−9.02)	−13.30 (−16.20–−9.90)	−16.55 (−21.98–−9.38)	−18.00 (−19.85–−15.60)	0.65
BAL (mean, SD)	−21.29 ± 12.53	−24.74 ± 12.18	−19.45 ± 15.80	−26.91 ± 12.64	−25.30 ± 6.38	0.679
GLS A2C (mean, SD)	−18.44 ± 4.42	−21.09 ± 6.54	−20.15 ± 3.81	−22.88 ± 4.38	−17.77 ± 0.97	0.077
BI (mean, SD)	−19.90 ± 15.21	−16.94 ± 7.82	−20.73 ± 4.30	−19.25 ± 8.34	−18.67 ± 4.03	0.804
MI (mean, SD)	−21.80 (−24.80–−20.40)	−26.15 (−30.65–−16.85)	−21.40 (−22.88–−19.38)	−25.05 (−28.95–−21.77)	−25.30 (−27.05–−21.65)	0.699
AI (mean, SD)	−30.10 (−34.40–−24.45)	−24.95 (−29.23–−20.53)	−24.55 (−28.93–−21.78)	−29.85 (−35.20–−22.85)	−20.00 (−22.80–−17.35)	0.209
AA (mean, SD)	−26.00 ± 12.49	−17.72 ± 5.75	−17.15 ± 7.19	−21.60 ± 8.32	−6.97 ± 7.22	0.081
MA (mean, SD)	−16.20 (−20.95–−11.55)	−17.30 (−22.73–−12.72)	−19.25 (−22.77–−15.55)	−18.00 (−23.73–−16.85)	−17.80 (−18.70–−11.20)	0.861
BA (mean, SD)	−19.90 (−27.10–−15.70)	−28.25 (−31.85–−14.97)	−21.05 (−26.57–−15.12)	−24.35 (−31.70–−18.85)	−17.50 (−27.30–−17.10)	0.923
GLS A3C (mean, SD)	−22.17 ± 2.88	−22.20 ± 6.28	−18.30 ± 1.56	−21.77 ± 5.26	−21.27 ± 8.22	0.566
BIL (mean, SD)	−35.39 ± 14.87	−30.04 ± 11.91	−24.07 ± 4.36	−23.62 ± 10.89	−22.17 ± 7.93	0.155
MIL (mean, SD)	−19.20 (−22.55–−12.35)	−23.90 (−28.68–−16.98)	−12.65 (−15.82–−9.18)	−18.20 (−25.67–−15.30)	−22.60 (−26.30–−22.15)	0.044
AIL (mean, SD)	−22.37 ± 8.11	−25.08 ± 9.02	−22.75 ± 4.06	−23.72 ± 9.15	−26.40 ± 13.08	0.907
AAS (mean, SD)	−23.57 ± 10.99	−24.25 ± 15.40	−22.12 ± 7.22	−23.93 ± 10.16	−27.03 ± 21.77	0.941
MAS (mean, SD)	−15.39 ± 8.39	−19.33 ± 7.83	−15.43 ± 4.29	−21.12 ± 6.95	−14.17 ± 4.37	0.043
BAS (mean, SD)	−9.13 ± 6.74	−15.43 ± 9.45	−13.70 ± 4.50	−19.34 ± 9.28	−18.77 ± 4.35	0.08

**Table 10:** Post hoc analysis of the BAV group according to phenotype (abbreviations: A3C—apical three-chamber view, MIL—mid-inferolateral, MAS—mid-anteroseptal).

GLS A3C MIL	*p*-Value	GLS A3C MAS	*p*-Value
0 AP vs. 0 LL	0.154	0 AP vs. 0 LL	0.181
0 AP vs. IA	0.315	0 AP vs. IA	0.776
0 AP vs. IB	0.481	0 AP vs. IB	0.103
0 AP vs. IC	0.183	0 AP vs. IC	0.833
0 LL vs. IA	0.029	0 LL vs. IA	0.143
0 LL vs. IB	0.229	0 LL vs. IB	0.589
0 LL vs. IC	0.875	0 LL vs. IC	0.146
IA vs. IB	0.051	IA vs. IB	0.099
IA vs. IC	0.057	IA vs. IC	0.858
IB vs. IC	0.317	IB vs. IC	0.068

## Data Availability

The original contributions presented in the study are included in the article; further inquiries can be directed to the corresponding author.

## References

[1] Zhang Y., Xiong T.-Y., Sondergard L., Mylotte D., Piazza N., Prendergast B., Chen M. (2023). Editorial: Bicuspid aortic valve: From pathophysiological mechanisms, imaging diagnosis to clinical treatment methods. Front. Cardiovasc. Med..

[2] Basso C. (2004). An echocardiographic survey of primary school children for bicuspid aortic valve. Am. J. Cardiol..

[3] Frye R.E., Ittleman B., Shabanova V., Sugeng L., Steele J., Ferdman D., Karnik R. (2023). Left ventricular strain in pediatric patients with bicuspid aortic valves and aortopathy. Prog. Pediatr. Cardiol..

[4] Mathieu P., Bossé Y., Huggins G.S., Della Corte A., Pibarot P., Michelena H.I., Limongelli G., Boulanger M.C., Evangelista A., Bédard E. (2015). The pathology and pathobiology of bicuspid aortic valve: State of the art and novel research perspectives. J. Pathol. Clin. Res..

[5] Henry M., Fadnes S., Lovstakken L., Mawad W., Mertens L., Nyrnes S.A. (2023). Flow Dynamics in Children with Bicuspid Aortic Valve: A Blood Speckle Tracking Study. Ultrasound Med. Biol..

[6] Ghiragosian C., Harpa M., Puscas A., Balau R., Al-Hussein H., Ghiragosian-Rusu S.E., Avram C., Baba D.F., Neagoe R., Suciu H. (2024). Histidine-Tryptophan-Ketoglutarate Cardioplegia Yields Different Results in Aortic Valve Surgery Depending on Patient Gender: A Pilot Study. Cureus.

[7] Anastasiou V., Daios S., Bazmpan M.A. (2023). Shifting from Left Ventricular Ejection Fraction to Strain Imaging in Aortic Stenosis. Diagnostics.

[8] Van der Ende J., Antona C.A.V., Orellana J.E., Cárdenas R., Roldan F.J., Barrón J.V. (2013). Left Ventricular Longitudinal Strain Measured by Speckle Tracking as a Predictor of the Decrease in Left Ventricular Deformation in Children with Congenital Stenosis of the Aorta or Coarctation of the Aorta. Ultrasound Med. Biol..

[9] Cheung Y.F. (2022). Echocardiographic strain imaging: What do paediatric cardiologists need to know?. Pediatr. Med..

[10] Sonaglioni A., Esposito V., Caruso C., Nicolosi G.L., Bianchi S., Lombardo M., Gensini G.F., Ambrosio G. (2021). Chest conformation spuriously influences strain parameters of myocardial contractile function in healthy pregnant women. J. Cardiovasc. Med..

[11] Levy P.T., Machefsky A., Sanchez A.A., Patel M.D., Rogal S., Fowler S., Yaeger L., Hardi A., Holland M.R., Hamvas A. (2016). Reference Ranges of Left Ventricular Strain Measures by Two-Dimensional Speckle-Tracking Echocardiography in Children: A Systematic Review and Meta-Analysis. J. Am. Soc. Echocardiogr..

[12] Jashari H., Rydberg A., Ibrahimi P., Bajraktari G., Kryeziu L., Jashari F., Henein M.Y. (2015). Normal ranges of left ventricular strain in children: A meta-analysis. Cardiovasc. Ultrasound.

[13] Naji P., Shah S., Svensson L.G., Gillinov A.M., Johnston D.R., Rodriguez L.L., Grimm R.A., Griffin B.P., Desai M.Y. (2017). Incremental Prognostic Use of Left Ventricular Global Longitudinal Strain in Asymptomatic/Minimally Symptomatic Patients with Severe Bioprosthetic Aortic Stenosis Undergoing Redo Aortic Valve Replacement Circulation. Cardiovasc. Imaging.

[14] Butcher S.C., Pio S.M., Kong W.K.F., Singh G.K., Ng A.C.T., Perry R., Sia C.H., Poh K.K., Almeida A.G., González A. (2022). Left ventricular remodelling in bicuspid aortic valve disease. Eur. Heart J. Cardiovasc. Imaging.

[15] Michelena H.I., Della Corte A., Evangelista A., Maleszewski J.J., Enriquez-Sarano M., Bax J.J., Otto C.M., Schäfers H.-J. (2020). Speaking a Common Language: Introduction to a Standard Terminology for the Bicuspid Aortic Valve and Its Aortopathy. Prog. Cardiovasc. Dis..

[16] Lopez L., Saurers D.L., Barker P.C., Cohen M.S., Colan S.D., Dwyer J., Forsha D., Friedberg M.K., Lai W.W., Printz B.F. (2024). Guidelines for Performing a Comprehensive Pediatric Transthoracic Echocardiogram: Recommendations from the American Society of Echocardiography. J. Am. Soc. Echocardiogr..

[17] Cantinotti M., Giordano R., Scalese M., Murzi B., Assanta N., Spadoni I., Maura C., Marco M., Molinaro S., Kutty S. (2017). Nomograms for Two-Dimensional Echocardiography Derived Valvular and Arterial Dimensions in Caucasian Children. J. Cardiol..

[18] R Core Team (2024). R: A Language and Environment for Statistical Computing.

[19] Făgărășan A., Săsăran M., Gozar L., Toma D., Șuteu C., Ghiragosian-Rusu S.-E., Al-Akel F.-C., Szabo B., Huțanu A. (2024). Circulating Matrix Metalloproteinases for Prediction of Aortic Dilatation in Children with Bicuspid Aortic Valve: A Single-Center, Observational Study. Int. J. Mol. Sci..

[20] Baumgartner H., Hung J., Bermejo J., Chambers J.B., Evangelista A., Griffin B.P., Iung B., Otto C.M., Pellikka P.A., Quiñones M. (2009). Echocardiographic Assessment of Valve Stenosis: EAE/ASE Recommendations for Clinical Practice. J. Am. Soc. Echocardiogr..

[21] Vahanian A., Beyersdorf F., Praz F., Milojevic M., Baldus S., Bauersachs J., Capodanno D., Conradi L., De Bonis M., De Paulis R. (2022). 2021 ESC/EACTS Guidelines for the Management of Valvular Heart Disease: Developed by the Task Force for the Management of Valvular Heart Disease of the European Society of Cardiology (ESC) and the European Association for Cardio-Thoracic Surgery (EACTS). Eur. Heart J..

[22] Pulapaka A.V., Giacone H.M. (2024). Giacone, Protecting Young Hearts: Sports Clearance for Young Patients at Risk for Sudden Cardiac Death. Curr. Pediatr. Rep..

[23] Senekovič Kojc T., Marčun Varda N. (2022). Novel Biomarkers of Heart Failure in Pediatrics. Children.

[24] Kusner J.J., Brown J.Y., Gleason T.G., Edelman E.R. (2023). The Natural History of Bicuspid Aortic Valve Disease. Struct. Heart.

[25] Hjertaas J.J., Einarsen E., Gerdts E., Kokorina M., Moen C.A., Urheim S., Saeed S., Matre K. (2023). Impact of aortic valve stenosis on myocardial deformation in different left ventricular levels: A three-dimensional speckle tracking echocardiography study. Echocardiography.

[26] Spaziani G., Girolami F., Arcieri L., Calabri G.B., Porcedda G., Di Filippo C., Surace F.C., Pozzi M., Favilli S. (2022). Bicuspid Aortic Valve in Children and Adolescents: A Comprehensive Review. Diagnostics.

[27] Baba D.F., Suciu H., Avram C., Danilesco A., Moldovan D.A., Rauta R.C., Huma L., Sin I.A. (2023). The Role of Preoperative Chronic Statin Therapy in Heart Transplant Receipts—A Retrospective Single-Center Cohort Study. Int. J. Environ. Res. Public Health.

[28] Ward R.M., Marsh J.M., Gossett J.M., Rettiganti M.R., Collins R.T. (2018). Impact of bicuspid aortic valve morphology on aortic valve disease and aortic dilation in pediatric patients. Pediatr. Cardiol..

[29] Liu T., Xie M., Lv Q., Li Y., Fang L., Zhang L., Deng W., Wang J. (2019). Bicuspid Aortic Valve: An Update in Morphology, Genetics, Biomarker, Complications, Imaging Diagnosis and Treatment. Front. Physiol..

[30] Verma S., Siu S.C. (2014). Aortic dilatation in patients with bicuspid aortic valve. N. Engl. J. Med..

[31] Yamauchi M.S.W., Puchalski M.D., Weng H.T., Pinto N.M., Etheridge S.P., Presson A.P., Tani L.Y., Minich L.L., Williams R.V. (2018). Disease progression and variation in clinical practice for isolated bicuspid aortic valve in children. Congenit. Heart Dis..

[32] Blais S., Meloche-Dumas L., Fournier A., Dallaire F., Dahdah N. (2020). Long-term risk factors for dilatation of the proximal aorta in a large cohort of children with bicuspid aortic valve. Circ. Cardiovasc. Imaging.

[33] Jashari H., Rydberg A., Ibrahimi P., Bajraktari G., Henein M.Y. (2015). Left ventricular response to pressure afterload in children: Aortic stenosis and coarctation: A systematic review of the current evidence. Int. J. Cardiol..

[34] Chatterjee S., Mukherjee S., Rani N., Kumar P., Kumar P., Sarkar A. (2022). Assessment of Cardiac Function in Children by Strain Imaging and its Correlation with Conventional Echocardiographic Parameter. Ann. Card. Anaesth..

[35] Carlos T., Freitas A.A., Alves P.M., Martins R., Gonçalves L. (2021). Aortic strain in bicuspid aortic valve: An analysis. Int. J. Cardiovasc. Imaging.

[36] Godlewski K., Dryżek P., Sadurska E., Werner B. (2021). Left ventricular systolic function impairment in children after balloon valvuloplasty for congenital aortic stenosis assessed by 2D speckle tracking echocardiography. PLoS ONE.

[37] Salcedo E.E., Gill E.A. (2021). Clinical Applications of Strain Imaging in Aortic Valve Disease.

[38] Alashi A., Mentias A., Abdallah A., Feng K., Gillinov A.M., Rodriguez L.L., Johnston D.R., Svensson L.G., Popovic Z., Griffin B.P. (2018). Incremental prognostic utility of left ventricular global longitudinal strain in asymptomatic patients with significant chronic aortic regurgitation and preserved left ventricular ejection fraction. JACC Cardiovasc. Imaging.

[39] Lang R.M., Badano L.P., Mor-Avi V., Afilalo J., Armstrong A., Ernande L., Flachskampf F.A., Foster E., Goldstein S.A., Kuznetsova T. (2015). Recommendations for cardiac chamber quantification by echocardiography in adults: An update from the American Society of Echocardiography and the European Association of Cardiovascular Imaging. J. Am. Soc. Echocardiogr..

[40] Marigliano A.-N., Ortiz J.-T., Casas J., Evangelista A. (2024). Aortic Regurgitation: From Valvular to Myocardial Dysfunction. J. Clin. Med..

[41] Menting M.E., van Grootel R.W.J., Bosch A.E.v.D., Eindhoven J.A., McGhie J.S., Cuypers J.A.A.E., Witsenburg M., Helbing W.A., Roos-Hesselink J.W. (2016). Quantitative assessment of systolic left ventricular function with speckle-tracking echocardiography in adult patients with repaired aortic coarctation. Int. J. Cardiovasc. Imaging.

